# Mycotoxins and neuropsychiatric symptoms: possible role in special refugee populations

**DOI:** 10.3389/fphar.2025.1524152

**Published:** 2025-03-19

**Authors:** Malvina Hoxha, Mariagrazia Abbasciano, Giuseppina Avantaggiato, Bruno Zappacosta, Domenico Tricarico

**Affiliations:** ^1^ Department for Chemical-Toxicological and Pharmacological Evaluation of Drugs, Faculty of Pharmacy, Catholic University Our Lady of Good Counsel, Tirana, Albania; ^2^ Institute of Sciences of Food Production (ISPA), National Research Council (CNR), Bari, Italy; ^3^ Department of Pharmacy and Pharmaceutical Sciences, University of Bari “Aldo Moro”, Bari, Italy; ^4^ Department of Biomedical Sciences, Faculty of Medicine, Catholic University Our Lady of Good Counsel, Tirana, Albania

**Keywords:** refugee populations, mycotoxins, neuropsychiatric symptoms, depression, anxiety

## 1 Introduction

In recent years, there has been a growing interest in assessing the role of mycotoxins in different diseases, especially in neurological disorders, and neuropsychiatric symptoms (Ratnaseelan et al., 2018; Nguyen et al., 2021; Abia et al., 2025; Ehsanifar et al., 2023). Mycotoxins are secondary metabolites produced by mold or fungi that contaminate food, and are responsible of biochemical and pathological changes in animals and humans. They can be found especially in cereals, wheat, coffee beans, maize, dried fruits (nuts, almonds, peanuts), dry-cured meat products, spices, rice, and crude vegetable oils (Pleadin et al., 2019). Mycotoxins can also be present in food even in the absence of visible molds, and industrial processing has little effect on their reduction. Moisture content, temperature, humidity, pH, physical damage of the food, are some of the factors that affect the mycotoxin biosynthesis (Richard, 2007). Their elimination from the body depends on the mycotoxin type, and varies from days to years. Controlling every step and condition during food production and storage process is essential in reducing the impact of these food safety hazard in human health. Ochratoxin A (OTA), patulin (PAT), aflatoxins, zearalenone (ZEA), trichothecenes, fumonisins are some of the most common mycotoxins found in food that can be involved in neuropsychiatric symptoms, and in neurological disorders (Von Tobel et al., 2014; Brand et al., 2019; Dai et al., 2024; Kowalska et al., 2016; Feuerstein et al., 2017; Chen et al., 2021).

Refugee populations, a group of individuals that cross the international border, and often live in refugee camps, temporary shelters in host countries, or urban areas, are more vulnerable to mycotoxins exposure. Refugees often rely on humanitarian aid for food, and often face resource constraints. In refugee settlements, humanitarian agencies consistently assess for major mycotoxins, such as ochratoxins, aflatoxins, and fumonisins, and reject crops with unsafe levels to protect refugees from exposure. Nevertheless, not all food lots can be tested due to resource limitations, and mycotoxins levels can increase after testing. This may occur during transit or storage, particularly under suboptimal conditions, before the food is prepared for meals. These challenges reveal the need for enhanced monitoring systems, better storage techniques, and robust protocols to ensure food safety throughout the supply chain. By addressing these challenges and implementing practical solutions, humanitarian agencies can more securely safeguard the wellbeing of refugee populations, reducing the risk of neuropsychiatric symptoms associated with exposure to mycotoxins.

The aim of this opinion paper is to discuss the potential role of mycotoxins in neuropsychiatric symptoms in vulnerable populations, such as refugees, who are particularly susceptible to food contamination and prolonged exposure to humid environments—factors that promote mold growth and mycotoxin production. Despite evidence linking mycotoxins to neuropsychiatric symptoms, research on their impact in refugee populations are deficient. This study addresses this gap by evaluating the potential role of mycotoxins exposure in refugees population, their neurological and psychiatric effects, and potential mitigation strategies. Different studies should be conducted to prove this hypothesis, taking in consideration also many confounding factors, such trauma, malnutrition, and chronic stress that might exacerbate the neuropsychiatric consequences of mycotoxin exposure.

## 2 Mycotoxins and neuropsychiatric symptoms

Exposure to mycotoxins is associated with a higher risk of cognitive impairment, depression, anxiety, and other neuropsychiatric symptoms (Ratnaseelan et al., 2018). Different studies have reported many neurological symptoms mycotoxin-associated, such as short-term memory loss, alteration of blink-reflex latency, color discrimination issues (Kilburn, 2003). In a study carried out in 182 subjects exposed to mold, moderate to severe levels of depression, and other physical, cognitive and emotional problems were reported. Patients were characterized by a hypoactivation in the frontal cortex (Crago et al., 2003). The presence of mold was also associated with emotional distress and depression in other studies (Hyndman, 1990; Packer et al., 1994).

The depletion of dopamine in mice treated with the OTA mycotoxin may also contribute to an increased risk of anxiety and depression (Pei et al., 2021). In addition, OTA can impair the hippocampal neurogenesis *in vivo* leading to cognitive impairment, difficulty in learning and concentration, and memory loss. Different studies report that OTA contributes to neurodegenerative disorders through DNA damage, oxidative stress, and mitochondrial dysfunction (Pei et al., 2021; Yoon et al., 2009; Zhang et al., 2009; Mateo et al., 2022). Long-term exposure to OTA can reduce cognitive function and cause attention deficits (Mateo et al., 2022).

In addition, fumonisin B1 (FB1) has been shown to have neurotoxic effects, inhibiting neurodevelopment, with increased levels observed in children with neural tube defects (NTD) and autism spectrum disorder (ASD) (Stockmann-Juvala and Savolainen, 2008; Ratnaseelan et al., 2018; Missmer et al., 2006; Yli-Mattila and Sundheim, 2022b). FB1 also suppresses the immune system, increasing the susceptibility to pathogens, and changing the expression of cytokine (Bulder et al., 2012; Wild et al., 2015). A chronic exposure to FB1can bring to different psychotic symptoms, such as memory disorders, and hallucinations (Chen et al., 2021).

PAT is a mycotoxin primarily found in apples and apple products, but also present in moldy fruits like grapes and pears, and has demonstrated neurotoxicity in various animal models (Song et al., 2014; Sabater Vilar et al., 2004). Furthermore, PAT is associated with nerve cell damage, irritability, confusion, alteration of communication between neurons, and uncoordinated movement (Pal et al., 2017; Brand et al., 2019).

Aflatoxins are one of the most important classes of mycotoxins, found in agricultural crops and comprising 21 members, all of which are capable of disrupting the blood-brain barrier. Alfatoxin B1 (AFB1) the most toxic member of the family, that has been involved in neurotoxicity, can cause depression, anxiety, memory deficit, and learning disorders (Dai et al., 2024; Qureshi et al., 2015). Animal models have shown that AFB1 affects neurotransmitter and neuropeptide regulation, leading to neurobehavioral abnormalities.

ZEA is another mycotoxin, that resembles estrogen and disrupts the hormone balance leading to irritability, and fluctuating mood (Kowalska et al., 2016).

Citrinin (CIT) is a mycotoxin found in wheat, rice, cheese, fermented sausages that can cause neurotoxic effects associated with damage in brain cells. Sleep deprivation, and anxiety are some of the symptoms that CIT can cause (Zargar and Wani, 2023a; Zargar and Wani, 2023b).

Trichothecenes are another class of mycotoxins that can cause neuroinflammation, leading to symptoms such as depression and headaches (Feuerstein et al., 2017). In particular, T-2 toxin, which is a member of trichothecene involved in the changes in neurotransmitters levels, can lead to cognitive impairment and increased irritability (Zhang et al., 2020a; Zhang et al., 2020b).

## 3 Discussion

Many studies have reported the role of mycotoxins in low- and middle-income countries, highlighting the need to evaluate intervention measures to prevent fatalities from acute mycotoxin intoxication, as well as the long-term effects from sub-acute exposure. In light of the above mentioned findings, mycotoxins can increase the vulnerability of neuropsychiatric symptoms in patients with already a higher risk, by accelerating the underlying neurologic, or pathologic processes (Empting, 2009).

Emotional distress, anxiety and depression, are also some of the neuropsychiatric symptoms frequent in special vulnerable populations, with particular need for help, such as in refugee populations. FB1 and ZEA levels were reported to be associated with drought stress in South Eastern Africa, sub-Saharan Africa, regions that host significant refugee populations (Yli-Mattila and Sundheim, 2022a; Pestka, et al., 1987; Ren et al., 2016a; Ren et al., 2016b; Ratnaseelan et al., 2018). Although the number of studies on mycotoxin levels and their effects on health has rapidly increased in recent years, there is a lack of research and review articles in current literature databases regarding mycotoxin serum and urine levels in refugee populations worldwide. In this context, measuring the levels of mycotoxins in different refugees populations around the world, such as in Africa, Middle East, or Southeast Asia would be of great interest, not only for studying their effects on health, but especially in neuropsychiatric disorders. Considering the pivotal role of mycotoxins in the high levels of stunting in children, we believe it would be of great public health interest to study their levels in these vulnerable populations as well.

We must also consider the numerous challenges, including the diversity of refugee populations, cultural contexts, national laws, and the lack of country-specific resources and data. It is also important to acknowledge the significant role of malnutrition, dietary deficiencies, psychological trauma, and post-traumatic stress disorder (PTSD) in contributing to neuropsychiatric symptoms in refugee populations. Deficiencies in vitamins (particularly vitamin B12), omega-3 fatty acids, and minerals, resulting from restricted access to adequate nutrition, are another contributing factor to neuropsychiatric symptoms in refugee populations, including mood disorders, anxiety, depression, and cognitive decline (Ogbu et al., 2022; Mason et al., 2012). In addition, many displaced individuals go through loss, violence, and experience an extreme emotional stress, or PTSD, which brings to manifestations of mental health disorders, including emotional instability, and social disengagement (Peconga and Høgh Thøgersen, 2020). Thus, it is essential to consider how trauma, PTSD, and malnutrition, may intersect with the role of mycotoxin exposure in neuropsychiatric outcomes.

Moreover, environmental pollutants like heavy metals (mercury, arsenic, lead) and pesticides may worsen neuropsychiatric symptoms in refugee populations, as they often live in areas with improper waste handling, and exposure to industrial pollutants that contaminate air, water, and food sources. The role of heavy metals in emotional disturbances, increased risk of depression and anxiety is widely acknowledged (Daher et al., 2025). On the other hand, in refugee populations, due to adjacency to farming activities, the exposure to pesticides combined with trauma and malnutrition, can exacerbate mental health issues (Richardson et al., 2019; Blackmore et al., 2020; Kamel et al., 2004).

Adapting monitoring campaigns to identify the most efficient and cost-effective strategies for assessing and reducing mycotoxin exposure in refugee populations is crucial. Efforts such as improving food storage, diversifying diets, and utilizing biomarkers of exposure can significantly reduce mycotoxin-related risks. These measures are essential for reducing neuropsychiatric symptoms associated with mycotoxin exposure and alleviating the suffering of affected populations.

Through this “opinion” study we propose an investigation of the levels of mycotoxins in these special populations, that will bring to further recommendations for the governmental and nongovernmental organizations, for either monitoring the prevalence of mycotoxins levels in neuropsychiatric symptoms in refugee populations, or for implementing various strategies to reduce mycotoxins exposure and contamination.
